# Influence of traditionality and modernity on public breastfeeding behaviors: a theory of planned behavior approach

**DOI:** 10.1186/s13006-025-00761-1

**Published:** 2025-08-21

**Authors:** Lin Cheng, Wen-Chi Wu, Yihjin Jin Hu, Chih Chien Cheng

**Affiliations:** 1 Department of Health Promotion and Health Education, National Taiwan Normal University, Taipei, Taiwan; 2 Department of Nursing, Hsin Sheng Junior College of Medical Care and Management, Taoyuan, Taiwan; 3 School of Medicine, College of Medicine, Fu Jen Catholic University, New Taipei City, Taiwan

**Keywords:** Breastfeeding in public, Traditionality, Modernity, Theory of planned behavior

## Abstract

**Background:**

Breast milk is widely recognized as the optimal source of infant nutrition, with the World Health Organization recommending exclusive breastfeeding for the first six months. However, breastfeeding rates remain suboptimal worldwide and in Taiwan. The six-month exclusive breastfeeding rate in Taiwan, defined as the percentage of infants aged six months who received only breast milk and no other foods or liquids in the previous 24 h, declined from 46.2% in 2018 to 37.9% in 2020. One barrier to continued breastfeeding is the discomfort or hesitation some mothers feel when breastfeeding in public. While qualitative research has indicated that conservative values may inhibit public breastfeeding, few quantitative studies have examined the simultaneous influence of traditionality and modernity. This study investigates how traditionality and modernity affect public breastfeeding behavior in Taiwan, using the Theory of Planned Behavior (TPB) as a framework.

**Methods:**

A cross-sectional online survey was conducted from 4 to 15 March 2024, recruiting 358 Taiwanese mothers with prior breastfeeding experience via social media. Validated instruments measured traditionality, modernity, TPB constructs (attitudes, subjective norms, perceived behavioral control, and intention), and frequency of public breastfeeding. Data were analyzed using descriptive statistics, bivariate correlations, and structural equation modeling.

**Results:**

Traditionality was negatively associated with attitudes (β = -0.30), perceived control (β = -0.11), intention (β = -0.14), and public breastfeeding behavior (β = -0.14). Modernity indirectly promoted public breastfeeding through positive links with attitudes (β = 0.12), subjective norms (β = 0.23), and perceived control (β = 0.26). Intention strongly predicted behavior (β = 0.60). The model explained 42% of the variance (CFI = 0.99, SRMR = 0.05).

**Conclusions:**

The study confirms that traditionality may hinder public breastfeeding, while modernity enhances it by shaping attitudes, norms, and control beliefs. These findings have important implications for public health policy and practice. Since traditionality and modernity influence behavior through distinct psychological pathways, interventions should be culturally tailored. For mothers with high traditionality, mobilizing family and community support may reinforce positive norms. For those with strong modern values, strategies should focus on autonomy, self-efficacy, and informed decision-making to support breastfeeding in public spaces.

**Supplementary Information:**

The online version contains supplementary material available at 10.1186/s13006-025-00761-1.

## Background

Breast milk is widely recognized as the optimal source of infant nutrition, with the World Health Organization recommending exclusive breastfeeding for the first six months [1]. However, rates remain suboptimal globally and in Taiwan, where the six-month exclusive breastfeeding rate (defined as the percentage of infants aged six months who received only breast milk and no other foods or liquids in the previous 24 h) declined from 46.2% in 2018 to 37.9% in 2020 [2]. One often overlooked but significant factor contributing to early cessation of breastfeeding is mothers’ reluctance to breastfeed in public. Despite legal protections such as Taiwan’s 2019 Public Breastfeeding Act [3], many mothers experience discomfort or social disapproval when breastfeeding in public spaces, including restaurants and public transport. These negative experiences, shaped by social norms and media portrayals, may discourage public breastfeeding and contribute to early cessation [4, 5], underscoring the need to understand the sociocultural influences, namely traditionality and modernity, that may affect breastfeeding behavior in public settings.

### Public breastfeeding

Public breastfeeding refers to directly nursing an infant at the breast, excluding bottle-feeding of expressed milk, in public spaces such as cafes, transportation hubs, or government buildings [6]. While legally protected in countries like the U.S., Australia, Norway, and Taiwan, breastfeeding in public remains a controversial and emotionally charged act. Some mothers have been denied the right to breastfeed in places like cafes or galleries, while others face stares, judgmental comments, or even intervention from security or staff [5]. These experiences contribute to negative emotions such as anxiety, embarrassment, or confusion [6], which may deter mothers from breastfeeding outside the home. Consequently, public breastfeeding plays a crucial role in shaping overall breastfeeding duration and deserves closer examination [5].

### Traditionality and modernity

The contrast between legal support and societal attitudes toward public breastfeeding in Taiwan reflects the ongoing tension between traditional and modern cultural values. Although breastfeeding in public is legally protected, many mothers still face embarrassment, scrutiny, and unfriendly environments [3]. A 2023 incident in Taiwan involving verbal abuse of a breastfeeding woman on a train illustrates the limited societal acceptance of public breastfeeding [7]. These reactions are rooted in traditional Chinese beliefs that regard breast exposure as morally inappropriate [8]. At the same time, Western values promoting gender equality and bodily autonomy have gradually influenced Taiwanese society, contributing to evolving norms around motherhood and breastfeeding [9].

This cultural shift can be better understood within the broader East Asian context. Psychological traits in the region are often shaped by collectivist and Confucian traditions, which emphasize obedience, family loyalty, and gender role conformity [10]. In contrast, modernity, shaped by Western ideals, prioritizes personal autonomy, gender equality, and emotional expression. Individuals in contemporary Taiwan often embody both orientations, forming a “bicultural self” that blends traditional and modern values [11]. Empirical studies have shown that traditionality is associated with more conservative gender role beliefs and greater discomfort with public breastfeeding. In contrast, modernity aligns with increased independence and support for maternal rights [12]. For example, research has found that Chinese women with high levels of traditionality view public breastfeeding as morally questionable [13].

In Taiwan, as feminist and individualist ideals become more prevalent, women may feel more confident breastfeeding in public without fear of social judgment [14, 15]. However, many continue to be influenced by traditional moral customs that shape attitudes and perceived norms around public breastfeeding. This dual influence is also evident in mainland China. For instance, Zhang et al. found that traditional moral values and perceived social norms significantly shaped exclusive breastfeeding practices among Chinese mothers, highlighting the critical role of subjective norms and maternal attitudes in influencing breastfeeding behavior. Similarly, in Taiwan, women are often influenced by deeply rooted patriarchal beliefs, such as the notion that “The man works, the woman keeps the home,” which impose subtle yet pervasive constraints on breastfeeding in both private and public domains. Despite existing legal protections, public breastfeeding continues to be perceived as inappropriate or shameful by some, reflecting a persistent dissonance between cultural expectations and institutional support [15]. These observations underscore how traditionality and modernity, within Chinese-influenced cultural traditions, shape women’s breastfeeding choices and their ability to exercise bodily autonomy.

### Theory of planned behavior

The Theory of Planned Behavior (TPB) offers a useful framework for understanding public breastfeeding behavior, positing that actions are guided by attitudes, subjective norms, and perceived behavioral control [16]. Studies have linked negative attitudes and social disapproval to reduced rates and shorter durations of public breastfeeding, while social support and a sense of control facilitate the behavior [4, 17, 18]. However, recent research has noted that TPB pays limited attention to cultural context and may benefit from the integration of culturally specific constructs to improve its explanatory power [19, 20]. This study addresses that gap by incorporating traditionality and modernity as cultural orientations that influence TPB components, offering a more contextually grounded understanding of mothers’ decisions to breastfeed in public.

### The present study

Although some qualitative studies have suggested that traditional values influence women’s decisions to breastfeed in public [21], few have quantitatively examined the relationship between cultural orientation and public breastfeeding behavior. To address this gap, the present study adopts the Theory of Planned Behavior to investigate how traditionality and modernity among Taiwanese mothers relate to attitudes, subjective norms, perceived behavioral control, behavioral intentions, and public breastfeeding behavior. Guided by the TPB framework and existing literature, we hypothesized that traditionality and modernity would directly and indirectly influence public breastfeeding behavior through TPB constructs. We also expected that attitudes, subjective norms, and perceived behavioral control would predict behavioral intentions, which would be associated with actual public breastfeeding behavior. In addition, the study explored the effects of breastfeeding knowledge and employment status as additional predictors of intention and behavior.

## Methods

### Recruitment and eligibility of participants

Given the lack of a sampling frame of mothers with breastfeeding experience, we could not obtain a representative sample from a defined population list. Therefore, we chose to distribute our questionnaire through online platforms frequented by Taiwanese mothers, such as Facebook and Line groups, to reach our target population effectively. Data for this cross-sectional study were gathered through online questionnaires distributed across various social networks from 4 to 15 March 2024. These networks were frequently visited by Taiwanese mothers with either current or previous breastfeeding experience. Eight of the initial 366 questionnaires collected were discarded because the respondents either did not have children or had not breastfed, leaving 358 valid questionnaires for a completion rate of 98%. The respondents came from all 19 counties and cities in Taiwan, ensuring a geographically comprehensive sample.

Inclusion criteria of participants included Taiwanese women over 20 who were breastfeeding or had breastfed, were mentally competent, free from severe mental health issues or cognitive disabilities, and were able to complete a questionnaire in Chinese. Exclusion criteria were women with any absolute contraindications to breastfeeding, such as HIV or HTLV-1 infections, those undergoing cancer treatment, substance users, or long-term users of psychiatric drugs. Mothers whose infants had conditions like galactosemia or hypermethioninemia were also excluded, as were those unable to breastfeed due to complications during childbirth.

### Ethics approval and consent to participate

The online questionnaire began with an informed consent form outlining the study’s procedures, goals, and the inclusion and exclusion criteria. Consent was required under the declaration “I participate in this study willingly,” which was presented as an icon on the first page of the questionnaire. Participants who clicked the icon were eligible to continue the questionnaire. Participants were free to withdraw at any moment without penalty, particularly in instances of discomfort or reluctance to answer questions. Participants who completed the questionnaire could win one of ten 100 NTD (approximately 3.34 USD) convenience store gift cards. The National Taiwan Normal University Research Ethics Committee approved the study’s methodology, with approval number 202401HS028. Human Ethics and Consent to Participate declarations: not applicable.

### Measures

#### TPB’s variables of breastfeeding in public places

To understand Taiwanese mothers’ attitudes, intentions, and behaviors toward breastfeeding in public places, the research team developed a questionnaire based on the Theory of Planned Behavior (TPB) by Ajzen [22]. Five breastfeeding and public health experts evaluated the questionnaire to ensure its applicability, accuracy, and completeness regarding the study’s goals and framework. The Content Validity Index (CVI) was 0.99, calculated after the expert validity review. The questionnaire includes sections on attitudes towards breastfeeding in public places, subjective norms about public breastfeeding, perceived behavioral control over breastfeeding in public, intentions to breastfeed in public, actual breastfeeding behavior in public, and control variables, including knowledge and demographic factors.

A pilot study with 45 mothers tested the first draft of the questionnaire. Using the pilot data, the breastfeeding knowledge scale was analyzed by calculating the item difficulty index and item discrimination index [23]. Four items with a discrimination index lower than 0.01 were removed, resulting in an overall average discrimination index of 0.46. Further item analysis was conducted, including extreme group testing (Critical Ratio, CR) and homogeneity testing (item-total correlation and Cronbach’s α) [24]. One attitude item with a CR below 3 was deleted, and all other items met the required standards. The measurements of the five concepts of TPB are described below.

#### Attitudes toward breastfeeding in public places

The scale used to measure attitudes toward breastfeeding in public places was developed based on previous studies [4, 8, 25, 26]. This scale consisted of nine items that assessed an individual’s positive or negative evaluations of breastfeeding and expressing milk in public places. Sample items are: “To extend the health benefits of continued breastfeeding for infants, it is necessary to breastfeed or express milk in public places,” and “Wearing appropriate clothing (such as nursing clothes and nursing bras) can enhance comfort and privacy when breastfeeding in public places.” Responses are rated on a scale from 1 (*strongly disagree*) to 5 (*strongly agree*), with the total score being the sum of all item scores. Higher scores indicate a higher degree of agreement or support for public breastfeeding. Exploratory factor analysis identified a single factor, explaining 64.7% of the variance. The Cronbach’s alpha for the scale was 0.77.

#### Subjective norms of breastfeeding in public spaces

The scale used to measure subjective norms regarding breastfeeding in public places was developed based on prior research [1, 2]. It included seven items that gauge beliefs about whether significant others think they should breastfeed in public. Sample items include: “My partner is supportive of me breastfeeding in public” and “My parents accept me breastfeeding in public places.” Responses were rated from 1 (*strongly disagree*) to 5 (*strongly agree*), with higher scores indicating stronger perceived social support. Exploratory factor analysis identified a single factor, explaining 68.6% of the variance, with a Cronbach’s alpha of 0.92.

#### Perceived control of breastfeeding in public spaces

The scale used to measure perceived behavioral control of breastfeeding in public places was developed based on previous research [3, 5]. This scale primarily aimed to understand individual perceptions of the control and difficulty of breastfeeding in public places, specifically whether individuals feel that breastfeeding in public is easy or difficult. The scale consisted of six items, each with five response options, scored from 1 (*strongly disagree*) to 5 (*strongly agree*). Sample items include “I am confident that I can breastfeed or express milk in public places” and “I feel that breastfeeding or expressing milk in public places is easy.” The total score is the sum of all item scores. A higher score indicates a greater perceived control over public breastfeeding behaviors by respondents. Exploratory factor analysis identified a single factor, explaining 68.6% of the variance. The Cronbach’s alpha for this measure of perceived behavioral control was 0.80.

#### Intention to breastfeed in public spaces

The scale used to measure intentions regarding breastfeeding behavior in public places was developed based on previous research [5]. It consisted of four questions that assessed the likelihood of breastfeeding in public. Sample questions include: “Would you be willing to breastfeed or express milk while dining in a restaurant?” Responses were rated from 1 (*very unwilling*) to 5 (*very willing*), with higher scores indicating stronger intentions. Exploratory factor analysis identified a single factor, explaining 68.6% of the variance, with a Cronbach’s alpha of 0.84.

#### Behavior of breastfeeding in public spaces

The scale used to measure breastfeeding behavior in public places was developed based on prior research [27, 28]. This scale mainly aimed to understand individual mothers’ breastfeeding behaviors in public places. It consisted of four questions, each with five response options: 1 for “never,” 2 for “rarely,” 3 for “sometimes,” 4 for “often,” and 5 for “always.” Sample items are “Have you ever breastfed your baby or expressed milk while dining in a restaurant?” and “Have you ever breastfed your baby or expressed milk while using public transportation?” The total score is the sum of all item scores. A higher score indicates a higher frequency of breastfeeding in public places by the respondents. Exploratory factor analysis identified a single factor, explaining 64.9% of the variance. The Cronbach’s alpha for this behavior scale was 0.81. The full questionnaire used in this study is provided in Additional file 1.

All constructs were assessed for convergent and discriminant validity. Average variance extracted (AVE) was used to examine convergent validity. While only the subjective norm construct exceeded the 0.50 threshold, the AVE values for other constructs were slightly lower but acceptable for exploratory research. Discriminant validity was evaluated by comparing the square root of each construct’s AVE with its correlations with other constructs. Most constructs met this criterion, except for intention and behavior, which showed conceptual overlap (square root of AVE for intention = 0.577 < *r* = 0.70). This finding aligns with the Theory of Planned Behavior, where intention is expected to be the closest predictor of actual behavior.

### Traditionality and modernity

The measurement of traditionality and modernity was based on the Chinese version of the “Multidimensional Individual Traditionality and Modernity Scale,” developed by Yang et al. [29]. This study employed a simplified version of this scale [30], which included 15 items for assessing traditionality and another 15 for modernity. The traditionality scale comprised five dimensions: obedience to authority, filial piety and ancestral reverence, contentment and compliance, fatalism and self-protection, and male superiority. The modernity scale encompasses five dimensions: equality and openness, independence and self-sufficiency, optimism and trust, respect for emotions, and gender equality. Each dimension was evaluated through three items, with response options ranging from 1 (strongly disagree) to 6 (strongly agree). The total scores of the two scales are the sum of the item scores of each scale. The reliability of the scales was confirmed with Cronbach’s alpha values of 0.81 for traditionality and 0.71 for modernity.

This study employed a simplified version of the ‘Multifaceted Individual Traditionality and Modernity Scale’ developed by Yang et al. [13]. Rooted in the Chinese cultural context, the scale is designed with high cultural sensitivity and relevance. It integrates traditional ideologies, such as Confucianism, Taoism, and Buddhism, alongside Western modern value systems, thereby capturing the psychological traits reflective of cultural transformation in East Asia. Given that Taiwanese society reflects a fusion of traditional and modern values, the scale is particularly well-suited to the sociocultural context of this study. In addition, the scale has been validated for reliability and construct validity in Taiwanese samples and has been widely adopted in related empirical research. Its use in this study enhances the cultural appropriateness of measurement and improves the interpretability and contextual relevance of the findings.

### Control variables

Factors associated with breastfeeding in public include maternal age, employment status, and knowledge about breastfeeding [27, 28, 31, 32, 33]. These factors were therefore included as control variables in the study. Age was divided into four categories: [1] 20–29 years [2], 30–39 years [3], 40–49 years, and [4] 50 years and over. Employment status was classified into employed and unemployed. The breastfeeding knowledge section consisted of eight questions with three answer choices: “Yes,” “No,” and “Don’t know.” Each correct answer earned 1 point, while incorrect or “Don’t know” answers received no points. The KR-20 value for the section on breastfeeding knowledge was 0.761. Scores ranged from 0 to 8, with higher scores indicating a higher level of knowledge about breastfeeding.

### Statistical analysis

Descriptive and bivariate analysis was conducted using SPSS 26. Spearman Correlation coefficients were used to explore the bivariate associations between variables. Path modeling and hypothesis testing were used with Amos version 24 to examine the direct and indirect associations of traditionality and modernity with the TPB concepts and public breastfeeding behavior.

## Results

### Sample characteristics

Table 1 shows the sample characteristics. Most participants were aged 30 to 39 (60.6%), followed by those 40 and older (24.3%) and those younger than 30 (15.1%). More than 70% of the mothers were employed (72.1%).

Analysis of the five main TPB variables indicated a moderate level of breastfeeding behavior and intention to breastfeed in public places, a relatively high level of attitudes preferring breastfeeding in public places, a moderate level of subjective norms favoring breastfeeding in public places, and a slightly higher level of perceived behavioral control (Table 1). The mean value of modernity was high (mean = 72.4), while traditionality showed a relatively low level (mean = 30.9). Participating mothers demonstrated a slightly high level of knowledge about breastfeeding.

**Table 1:** Sample characteristics (*N* = 358)

Variable	*n*	%		
Age				
20–29 years	54	15.1		
30–39 years	217	60.6		
40–49 years	63	17.6		
50 years and above	24	6.7		
Employment				
Unemployed	100	27.9		
Employed	258	72.1		
	*Min*	*Max*	*Mean*	*SD*
Breastfeeding in Public Places (Range: 4–20)	4	20	10.07	4.61
Intentions of Breastfeeding in Public Places (Range: 4–20)	4	20	15.24	3.75
Attitude (Range: 9–45)	17	45	34.59	4.97
Subjective Norms (Range: 7–35)	7	35	25.66	6.37
Perceived Behavioral Control (Range: 6–35)	11	30	24.08	4.37
Modernity (Range: 15–90)	40	90	72.37	8.86
Traditionality (Range: 15–90)	15	76	30.87	9.29
Breastfeeding Knowledge (Range: 0–8)	1	8	6.72	1.74

### Bivariate associations among main variables

The correlation coefficients among all study variables were less than 0.8, indicating no significant multicollinearity issues in this study [34] (Table 2). Regarding the associations of the five main variables in the TPB model, breastfeeding in public places, intentions, attitudes, subjective norms, and perceived behavioral control were positively correlated, and the correlations were higher than 0.5. Traditionality was significantly negatively associated with the main TPB variables besides subjective norms. Modernity was significantly associated with the five main TPB variables. Knowledge was positively associated with the five main TPB variables and modernity, and was negatively associated with traditionality.

**Table 2:** Spearman correlation coefficients among study variables

	1	2	3	4	5	6	7	8	9	10
1. Breastfeeding in Public Places	1									
2. Intentions of Breastfeeding	0.630^**^	1								
3. Attitudes	0.489^**^	0.604^**^	1							
4. Subjective Norms	0.348^**^	0.655^**^	0.499^**^	1						
5. Perceived Behavioral Control	0.463^**^	0.742^**^	0.558^**^	0.685^**^	1					
6. Modernity	0.126^*^	0.294^**^	0.224^**^	0.278^**^	0.336^**^	1				
7. Traditionality	− 0.323^**^	− 0.231^**^	− 0.319^**^	− 0.006	− 0.171^**^	− 0.266^**^	1			
8. Knowledge	0.233^**^	0.345^**^	0.324^**^	0.314^**^	0.354^**^	0.229^**^	− 0.163^**^	1		
9. Age	− 0.185^**^	− 0.208^**^	− 0.110^*^	− 0.152^**^	− 0.220^**^	− 0.254^**^	0.208^**^	− 0.074	1	
10. Employment	− 0.129^*^	0.024	− 0.051	0.107^*^	0.055	0.086	0.120^*^	− 0.142^**^	0.166^**^	1

*p < 0.05, **p < 0.01, ***p < 0.00

### Direct and indirect associations of traditionality and modernity with TPB variables

**Fig. 1:**
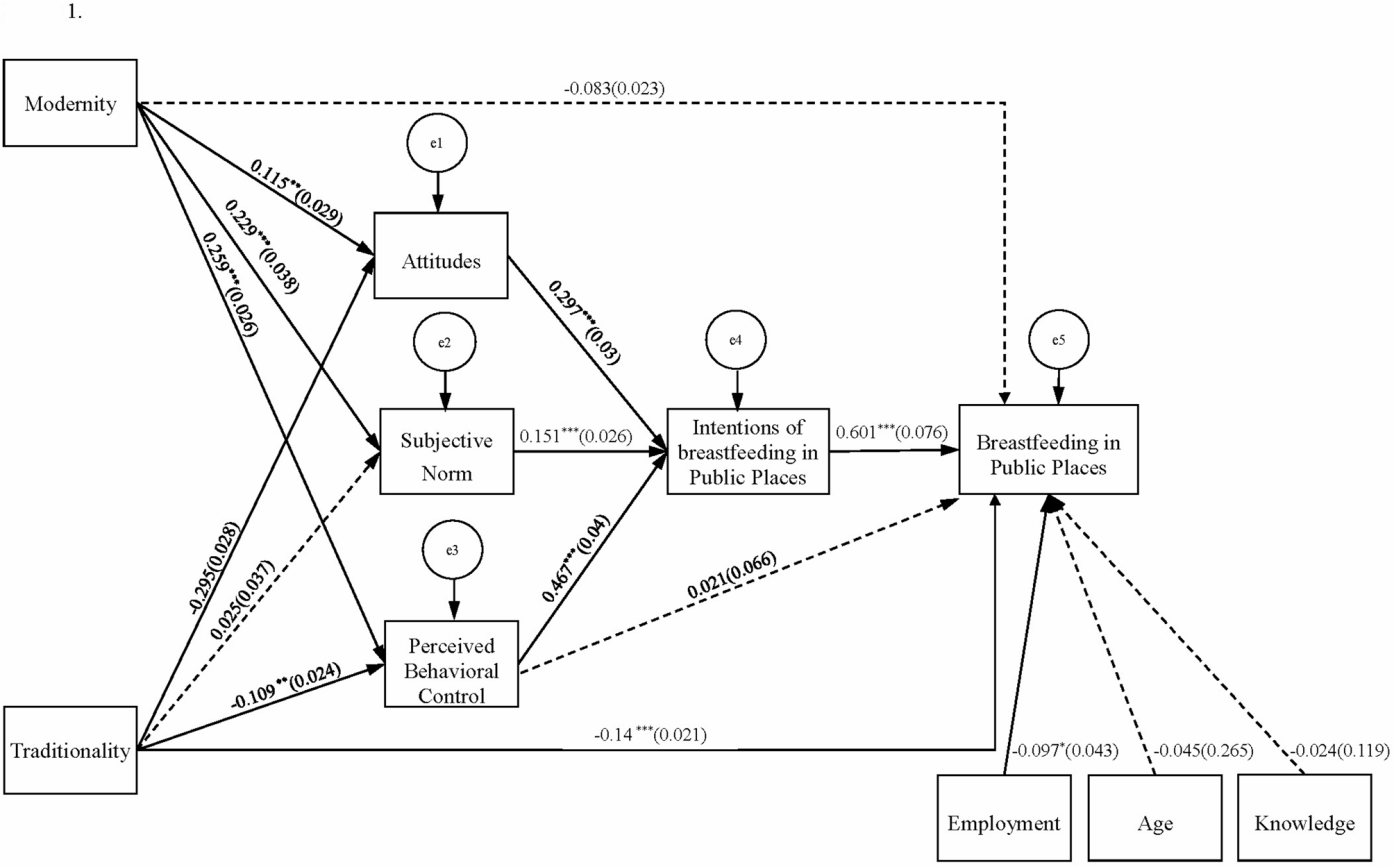
Final structural model showing direct and indirect effects among traditionality, modernity, TPB constructs, and public breastfeeding behavior

We constructed a structural model that included all observed study variables with significant correlations and drew paths based on the research framework. The final model (Fig. 1) demonstrates a good level of fit (χ² = 29.77, *df* = 15, χ²/*df* = 1.99, CFI = 0.99, and SRMR = 0.05), supporting the direct associations between modernity and traditionality with the predicted TPB variables. Relevant estimates are shown in Fig. 1; Table 3. The explained variances (R-square) for each variable in the model are as follows: attitude = 0.118, subjective norms = 0.05, perceived behavioral control = 0.094, behavioral intention = 0.637, and public breastfeeding behavior = 0.419.

The results indicate that traditionality significantly negatively affected public breastfeeding behavior (standardized beta, STB = -0.14, *p* < 0.001), suggesting that participants with higher levels of traditionality reported a lower frequency of breastfeeding in public. Traditionality was also negatively associated with attitudes (STB = -0.295, *p* < 0.001) and perceived behavioral control (STB = -0.109, *p* = 0.036), but showed no significant association with subjective norms (STB = -0.025, *p* = 0.638). Further analysis using bootstrapping (2,000 resamples) revealed that traditionality indirectly influenced behavioral intention to breastfeed in public through its effects on attitude, subjective norms, and perceived behavioral control (STB = -0.135, *p* < 0.001; 95% CI: [-0.209, -0.062]). In addition, traditionality had a significant indirect effect on public breastfeeding behavior through these same mediators (STB = -0.083, *p* < 0.001; 95% CI: [-0.132, -0.040]).

In contrast, modernity did not significantly affect public breastfeeding behavior (STB = -0.083, *p* = 0.062). However, participants with higher levels of modernity showed significantly more favorable attitudes (STB = 0.115, *p* = 0.026), stronger subjective norms (STB = 0.229, *p* < 0.001), and greater perceived behavioral control (STB = 0.259, *p* < 0.001), indicating positive associations with key TPB components. Further analysis using bootstrapping (2,000 resamples) revealed a significant indirect effect of modernity on public breastfeeding behavior mediated by these components. Specifically, modernity was positively associated with behavioral intention (STB = 0.190, *p* < 0.001; 95% CI: [0.097, 0.279]), and also had an indirect effect on public breastfeeding behavior through the same pathways (STB = -0.119, *p* < 0.001; 95% CI: [0.062, 0.179]). These findings suggest that although modernity does not directly influence public breastfeeding behavior, it plays a meaningful motivational role by strengthening attitudes, perceived social support, and self-efficacy related to breastfeeding in public.

Regarding the TPB core variables, higher levels of attitude (STB = 0.297, *p* < 0.001), subjective norms (STB = 0.151, *p* < 0.001), and perceived behavioral control (STB = 0.467, *p* < 0.001) were significantly associated with stronger behavioral intentions, which in turn were positively related to more frequent public breastfeeding behavior (STB = 0.601, *p* < 0.001). However, perceived behavioral control did not have a significant direct association with public breastfeeding behavior (STB = 0.021, *p* = 0.738). Among the control variables, employment status showed a significant negative direct effect on public breastfeeding behavior (STB = -0.097, *p* = 0.021), whereas age (STB = -0.045, *p* = 0.301) and breastfeeding knowledge (STB = -0.024, *p* = 0.593) were not significantly associated. Mediation analysis revealed that attitude indirectly influenced public breastfeeding behavior through behavioral intention (STB = 0.179, *p* < 0.001; 95% CI: [0.115, 0.242]). Similarly, subjective norms (STB = 0.091, *p* < 0.001; 95% CI: [0.026, 0.155]) and perceived behavioral control (STB = 0.281, *p* < 0.001; 95% CI: [0.203, 0.370]) also had significant indirect effects via intention.

**Table 3:** Standardized coefficients of the path model for breastfeeding behavior among sample mothers (*N* = 358)

	Dependent Variables														
Independent Variables	Attitudes			Subjective Norms			Perceived Behavioral Control			Intentions of breastfeeding in public places			Breastfeeding in Public Places		
	Direct	Indirect	Total	Direct	Indirect	Total	Direct	Indirect	Total	Direct	Indirect	Total	Direct	Indirect	Total
Traditionality	-0.294^***^		-0.295^***^	0.025		0.025	-0.109^**^		-0.109^**^		-0.135^***^	-0.135^***^	-0.140^***^	-0.083^***^	-0.223^***^
Modernity	0.115		0.115	0.229^***^		0.229^***^	0.259^***^		0.259^***^		0.190^***^	0.190^***^	-0.083	0.119^***^	0.360^***^
Attitude										0.297^***^		0.297^***^		0.179^***^	0.179^***^
Subjective Norms										0.151^**^		0.151^**^		0.091^**^	0.091^**^
Perceived Behavioral Control										0.467^***^		0.467^***^	0.021	0.281^***^	0.302^***^
Behavioral Intentions													0.601^***^		0.601^***^
Age ^a^													-0.045		-0.045
Employment ^b^													-0.097^*^		-0.097^*^
Knowledge													-0.024		-0.024
*R-square*		0.118			0.050			0.094			0.637			0.419	

*p < 0.05, **p < 0.01, ***p < 0.001

^a^ 1 = 20–29 years; 2 = 30–39 years; 3 = 40–49 years; 4 = 50 years and above

^b^ 1 = Unemployed; 2 = Employed

## Discussion

This study found that modernity did not directly influence public breastfeeding behavior, but had a significant indirect effect through attitudes, subjective norms, and perceived behavioral control, shaping behavioral intentions. Although correlation analysis showed a positive association between modernity and public breastfeeding, the direct path became nonsignificant in the structural model when mediating factors were considered. While prior research has not directly examined modernity as a whole in relation to public breastfeeding, studies focusing on its core components, namely gender equality, independence, emotional expression, and optimism, offer meaningful insights. Regarding gender equality, women who endorse egalitarian values are more likely to reject traditional gender roles and assert their right to breastfeed in public, as shown in findings by Augusto et al. [35]. Regarding independence, Qiu emphasized that postpartum women who prioritize autonomy tend to make informed decisions about breastfeeding, which is consistent with this study’s results [16]. Respect for emotional expression also plays a role: women who value emotional well-being are more likely to feel confident and proud when breastfeeding [36]. In public settings, however, external support—such as encouragement from family or positive social norms—may be essential to sustain such confidence [5, 18, 28]. Lastly, optimism has been linked to higher self-efficacy and continued breastfeeding, as found by Yadollahi et al. [21] and Brown [38]. In contrast, pessimism is associated with early cessation due to embarrassment and low confidence. These four components of modernity may indirectly influence breastfeeding behavior by shaping attitudes, subjective norms, and perceived behavioral control, the key mechanisms proposed by TPB that link individual characteristics to behavioral intention and, ultimately, to action.

In contrast to modernity, this study found that traditionality negatively influenced attitudes, perceived behavioral control, behavioral intentions, and the frequency of breastfeeding in public, and also showed a significant direct effect on public breastfeeding behavior. These findings can be better understood by examining the core components of traditionality, including obedience to authority, gender role conformity, modesty, and symbolic views of the female body. First, the emphasis on obedience to authority, reflected in items such as “when the couple has different opinions, the wife should obey the husband,” illustrates how traditional values uphold hierarchical family structures and behavioral norms in social contexts. This may limit women’s autonomy and reduce their confidence in breastfeeding publicly [26]. Second, gender role conformity is closely tied to the expectation that women should be modest and discreet. Women who strongly adhere to traditional values may avoid breastfeeding in public due to prevailing social expectations regarding modesty and propriety. Sheehan et al. [37] highlighted that cultural beliefs emphasizing feminine decorum can shape maternal attitudes and restrict public breastfeeding practices. Similarly, Zhang et al. [15] reported that mothers who perceive breastfeeding in public as morally questionable tend to experience greater discomfort and reduced self-efficacy. Such beliefs often stem from cultural norms that regard the exposure of the female body—even in the context of infant feeding—as inappropriate or socially unacceptable [28]. These perceptions may contribute to internalized stigma, which in turn reduces mothers’ perceived behavioral control over breastfeeding in public spaces. From a Symbolic Interactionist perspective, breastfeeding behaviors may carry layered social meanings. While breastfeeding is often associated with nurturing and maternal care, cultural and social norms can shape how these behaviors are perceived in public settings. For instance, psychosocial factors such as maternal self-efficacy, social support, and perceived expectations significantly influence breastfeeding intentions and practices [18, 37]. Moreover, intention, confidence, and a supportive environment are crucial for sustaining breastfeeding behavior [38]. These findings suggest that maternal decisions regarding public breastfeeding are influenced not only by individual beliefs but also by broader sociocultural contexts. This tension may elicit discomfort or fear of social judgment, potentially undermining maternal confidence and decreasing the likelihood of breastfeeding in public. Notably, the study found that individual traditionality had no significant impact on subjective norms, contradicting the original hypothesis. One plausible explanation is that traditional values are deeply internalized, making individuals less responsive to perceived social approval or disapproval. Moreover, the study assessed injunctive norms—such as support from significant others—but did not include descriptive norms, which reflect the perceived prevalence of public breastfeeding behaviors. Since individuals with strong traditional beliefs may be more attuned to observed behaviors than to verbal expressions of approval, traditionality may be more closely related to descriptive norms. Future research should examine this possibility and refine the operationalization of normative constructs within culturally specific applications of the Theory of Planned Behavior (TPB).

Consistent with previous research, this study supports the utility of the TPB framework in explaining public breastfeeding behavior. Attitudes, subjective norms, and perceived behavioral control were positively associated with behavioral intentions, which in turn significantly predicted the frequency of public breastfeeding, a finding consistent with Olejnik et al. (2022), who noted that behavior is mainly determined by intention, and intention is composed of attitudes and subjective norms [39]. Public attitudes have been shown to influence perceptions of acceptability surrounding breastfeeding in public spaces [40]. Supportive environments, such as encouragement from family members or workplace accommodations, can enhance maternal self-efficacy and motivation to breastfeed in public [18, 38]. The involvement of partners and extended family, such as grandparents or mothers-in-law, may further strengthen maternal intentions and alleviate anxiety [28, 41]. While perceived behavioral control was positively associated with intentions, it did not directly predict public breastfeeding behavior in this study, possibly due to its effect being primarily mediated through intention formation.

Several limitations of this study should be acknowledged. First, data were collected through an online questionnaire, which may have introduced self-selection bias. Participants who chose to complete the survey may have had stronger opinions or personal experiences related to breastfeeding in public, potentially limiting the diversity of perspectives captured. Second, convenience sampling limits the sample’s representativeness, which may affect the generalizability of the findings. Lastly, the cross-sectional design prevents causal inferences, capturing data at a single time point rather than tracking changes or developments over time.

In addition, this study extends the Theory of Planned Behavior (TPB) by incorporating the constructs of traditionality and modernity, offering a novel perspective on its application in culturally dynamic and socially evolving contexts. While TPB has been widely used to predict health behaviors, it has rarely been combined with broader cultural value systems as core explanatory variables. The findings of this study demonstrate that traditional and modern values can indirectly influence public breastfeeding behavior through their effects on attitudes, subjective norms, and perceived behavioral control. By addressing TPB’s limited sensitivity to cultural influences, this study highlights the model’s potential for greater adaptability and relevance in societies marked by cultural diversity and shifting social norms.

## Conclusions

The study confirms that traditionality may impede breastfeeding behavior in public places, while modernity can elevate it via enhancing attitudes, social norms, and behavioral controls of breastfeeding in public places.

The results of this study have important implications for public health policy and practice. Since traditionality and modernity influence public breastfeeding behavior through different psychological pathways, future interventions should be culturally tailored. For mothers with high traditionality, strategies may focus on mobilizing family and social support to reinforce positive norms around breastfeeding. In contrast, for mothers with strong modern values, efforts should emphasize autonomy, self-efficacy, and informed decision-making.

## Supplementary Information

Below is the link to the electronic supplementary material.

Supplementary Material 1: Additional file 1. Survey instrument – Questionnaire assessing traditionality, modernity, and TPB variables related to public breastfeeding

## Data Availability

The data are available upon request from the corresponding author.
