# Supplementary material for: Influence of traditionality and modernity on public breastfeeding behaviors: a theory of planned behavior approach
Source: Int Breastfeed J. 2025 Aug 21;20:66. doi: 10.1186/s13006-025-00761-1 (PMC12369124; doi:10.1186/s13006-025-00761-1)
Supplement: Supplementary file 1 — Supplementary Material 1: Additional file 1. Survey instrument – Questionnaire assessing traditionality, modernity, and TPB variables related to public breastfeeding [file 13006_2025_761_MOESM1_ESM.docx]

## Survey on Breastfeeding and Milk Expression in Public Places

Instructions:
When we refer to 'breastfeeding in public,' we mean a woman feeding her baby directly at the breast or expressing milk in public settings such as restaurants, parks, shopping malls, or public transportation, to meet the needs of both the mother and the infant. This may involve breastfeeding without additional privacy measures or using items like nursing clothes or nursing covers to partially or fully conceal the breast.

Before starting the questionnaire, may we ask if you are at least 20 years old?
□ Yes □ No

### Part 1: Attitudes Toward Public Breastfeeding

(Please respond based on your true thoughts and feelings.)
(1) Strongly Disagree (2) Disagree (3) Neutral (4) Agree (5) Strongly Agree

1. It is better for working mothers to feed their babies with formula. □ 1 □ 2 □ 3 □ 4 □ 5

2. Women should not breastfeed in public places (e.g., restaurants, stations, public transportation). □ 1 □ 2 □ 3 □ 4 □ 5

3. I think breastfeeding in public attracts unnecessary attention. □ 1 □ 2 □ 3 □ 4 □ 5

4. I would feel uncomfortable if a woman breastfeeds near me. □ 1 □ 2 □ 3 □ 4 □ 5

5. Breastfeeding should be done in private settings. □ 1 □ 2 □ 3 □ 4 □ 5

6. Wearing appropriate clothing (e.g., nursing clothes, nursing covers) improves comfort and privacy when breastfeeding in public. □ 1 □ 2 □ 3 □ 4 □ 5

7. To prolong the health benefits of breastfeeding, breastfeeding or expressing milk in public is necessary. □ 1 □ 2 □ 3 □ 4 □ 5

8. Breastfeeding in public reduces the burden of carrying supplies when going out. □ 1 □ 2 □ 3 □ 4 □ 5

9. Legislation protecting public breastfeeding has no direct impact on breastfeeding duration. □ 1 □ 2 □ 3 □ 4 □ 5

### Part 2: Social Acceptance of Public Breastfeeding

(1) Strongly Disagree (2) Disagree (3) Neutral (4) Agree (5) Strongly Agree

1. My partner would accept me breastfeeding in public. □ 1 □ 2 □ 3 □ 4 □ 5

2. My partner's parents would accept me breastfeeding in public. □ 1 □ 2 □ 3 □ 4 □ 5

3. My own parents would accept me breastfeeding in public. □ 1 □ 2 □ 3 □ 4 □ 5

4. My friends would accept me breastfeeding in public. □ 1 □ 2 □ 3 □ 4 □ 5

5. My relatives would accept me breastfeeding in public. □ 1 □ 2 □ 3 □ 4 □ 5

6. My supervisors and colleagues would accept me breastfeeding in public. □ 1 □ 2 □ 3 □ 4 □ 5

7. Maternal and child healthcare professionals would accept me breastfeeding in public. □ 1 □ 2 □ 3 □ 4 □ 5

### Part 3: Perceived Behavioral Control

(1) Strongly Disagree (2) Disagree (3) Neutral (4) Agree (5) Strongly Agree

1. I can decide for myself whether to breastfeed or express milk in public. □ 1 □ 2 □ 3 □ 4 □ 5

2. I am confident I can breastfeed or express milk in public. □ 1 □ 2 □ 3 □ 4 □ 5

3. I find it easy to breastfeed or express milk in public. □ 1 □ 2 □ 3 □ 4 □ 5

4. I am unable to breastfeed or express milk in public. □ 1 □ 2 □ 3 □ 4 □ 5

5. I prepare items like nursing covers for breastfeeding or expressing milk in public. □ 1 □ 2 □ 3 □ 4 □ 5

6. I know how to locate nursing rooms when needed. □ 1 □ 2 □ 3 □ 4 □ 5

### Part 4: Willingness to Breastfeed in Different Situations

(1) Very Unwilling (2) Unwilling (3) Neutral (4) Willing (5) Very Willing

1. When friends or relatives visit at home, would you continue breastfeeding or expressing milk during the gathering? □ 1 □ 2 □ 3 □ 4 □ 5

2. Would you be willing to breastfeed or express milk in a public nursing room? □ 1 □ 2 □ 3 □ 4 □ 5

3. Would you be willing to breastfeed or express milk while dining at a restaurant? □ 1 □ 2 □ 3 □ 4 □ 5

4. Would you be willing to breastfeed or express milk on public transportation (e.g., bus, MRT, train, airplane)? □ 1 □ 2 □ 3 □ 4 □ 5

### Part 5: Experience of Public Breastfeeding

(1) Never (2) Occasionally (3) Sometimes (4) Often (5) Always

1. Have you breastfed your baby while dining at a restaurant? □ 1 □ 2 □ 3 □ 4 □ 5

2. Have you breastfed your baby on public transportation? □ 1 □ 2 □ 3 □ 4 □ 5

3. Have you breastfed your baby in a public nursing room? □ 1 □ 2 □ 3 □ 4 □ 5

4. Have you continued breastfeeding during gatherings with visiting friends or family at home? □ 1 □ 2 □ 3 □ 4 □ 5

5. Have you expressed milk directly at your seat while dining at a restaurant? □ 1 □ 2 □ 3 □ 4 □ 5

6. Have you expressed milk directly at your seat while using public transportation? □ 1 □ 2 □ 3 □ 4 □ 5

7. Have you expressed milk in a public nursing room? □ 1 □ 2 □ 3 □ 4 □ 5

8. Have you continued expressing milk during gatherings at home? □ 1 □ 2 □ 3 □ 4 □ 5

### Part 6: Knowledge about Breastfeeding

(1) True (2) False (0) Don't Know

1. Most mothers produce enough milk to feed their babies. □ 1 □ 2 □ 3

2. Breastfed babies are healthier than formula-fed babies. □ 1 □ 2 □ 3

3. Breastfeeding helps prevent respiratory infections. □ 1 □ 2 □ 3

4. Breastfeeding reduces the risk of allergies. □ 1 □ 2 □ 3

5. Breastfeeding can start immediately after birth. □ 1 □ 2 □ 3

6. Breastfeeding reduces the risk of ear infections in infants. □ 1 □ 2 □ 3

7. Breastfeeding reduces the incidence of gastrointestinal infections. □ 1 □ 2 □ 3

8. Women who breastfeed have a lower risk of breast and ovarian cancer. □ 1 □ 2 □3

### Part 7: Personal Information

Age group: □ (1) 20–29 □ (2) 30–39 □ (3) 40–49 □ (4) 50–59 □ (5) 60 and above

Education level: □ (1) Junior High or below □ (2) High School/Vocational □ (3) College/University □ (4) Graduate School or above

Occupation: □ (1) Parental leave □ (2) Military/Police □ (3) Public Servant □ (4) Education □ (5) Business □ (6) Industry □ (7) Agriculture □ (8) Healthcare □ (9) Service Industry □ (10) Food & Beverage □ (11) Student □ (12) Unemployed □ (13) Other: _______

Current residence: ___________ County/City ___________ District

On a ladder from 1 to 10 representing the worst to the best life in Taiwan, where would you place your current life? □ 1 □ 2 □ 3 □ 4 □ 5 □ 6 □ 7 □ 8 □ 9 □ 10

Marital status: □ (1) Single □ (2) In a relationship, not married □ (3) Married □ (4) Divorced □ (5) Widowed

Feeding method for your youngest child: □ (1) No breast milk □ (2) Exclusively breastfed □ (3) Exclusively fed expressed milk □ (4) Mostly breastfed, occasionally expressed milk □ (5) Mostly expressed milk, occasionally breastfed □ (6) Received donor milk

### Part 8: Values and Beliefs

(1) Strongly Disagree (2) Disagree (3) Slightly Disagree (4) Slightly Agree (5) Agree (6) Strongly Agree

Traditional Values

1. Couples should have their birth charts checked before marriage to avoid incompatibility. □ 1 □ 2 □ 3 □ 4 □ 5 □ 6

2. Children should respect those their parents respect. □ 1 □ 2 □ 3 □ 4 □ 5 □ 6

3. When spouses disagree, the wife should obey the husband. □ 1 □ 2 □ 3 □ 4 □ 5 □ 6

4. Sending parents to nursing homes is an act of filial disrespect. □ 1 □ 2 □ 3 □ 4 □ 5 □ 6

5. Focusing only on self-protection is an important principle. □ 1 □ 2 □ 3 □ 4 □ 5 □ 6

6. Seniority should outweigh ability in workplace rewards. □ 1 □ 2 □ 3 □ 4 □ 5 □ 6

7. Disputes should be mediated by the eldest family member. □ 1 □ 2 □ 3 □ 4 □ 5 □ 6

8. The man should be the head of the household. □ 1 □ 2 □ 3 □ 4 □ 5 □ 6

9. A daughter-in-law should serve her parents-in-law. □ 1 □ 2 □ 3 □ 4 □ 5 □ 6

10. Wealth and success are determined by fate. □ 1 □ 2 □ 3 □ 4 □ 5 □ 6

11. The more young people know about sex, the more problems arise. □ 1 □ 2 □ 3 □ 4 □ 5 □ 6

12. Obeying authority and respecting elders are virtues children should learn. □ 1 □ 2 □ 3 □ 4 □ 5 □ 6

13. Women should not participate in political activities. □ 1 □ 2 □ 3 □ 4 □ 5 □ 6

14. Children should behave respectfully in front of elders. □ 1 □ 2 □ 3 □ 4 □ 5 □ 6

15. It is best to avoid arguments with powerful or wealthy individuals. □ 1 □ 2 □ 3 □ 4 □ 5 □ 6

Modern Values

1. Spouses of different religions should respect each other's beliefs. □ 1 □ 2 □ 3 □ 4 □ 5 □ 6

2. Adult children should manage their own earnings. □ 1 □ 2 □ 3 □ 4 □ 5 □ 6

3. A free-market system is essential for economic prosperity. □ 1 □ 2 □ 3 □ 4 □ 5 □ 6

4. Premarital sex is acceptable if both parties love each other. □ 1 □ 2 □ 3 □ 4 □ 5 □ 6

5. Women should have the same sexual freedom as men. □ 1 □ 2 □ 3 □ 4 □ 5 □ 6

6. Children should argue reasonably even if their parents disagree. □ 1 □ 2 □ 3 □ 4 □ 5 □ 6

7. Married children should not live with their parents. □ 1 □ 2 □ 3 □ 4 □ 5 □ 6

8. Everyone should receive as much education as possible. □ 1 □ 2 □ 3 □ 4 □ 5 □ 6

9. There is nothing wrong with marrying a divorced person. □ 1 □ 2 □ 3 □ 4 □ 5 □ 6

10. Husbands and wives should have their own friends. □ 1 □ 2 □ 3 □ 4 □ 5 □ 6

11. Divorce is a solution if marriage becomes too painful. □ 1 □ 2 □ 3 □ 4 □ 5 □ 6

12. A wife should decide for herself even if opinions differ. □ 1 □ 2 □ 3 □ 4 □ 5 □ 6

13. A good job enables learning new things. □ 1 □ 2 □ 3 □ 4 □ 5 □ 6

14. Husbands should not feel ashamed if their wives have higher achievements. □ 1 □ 2 □ 3 □ 4 □ 5 □ 6

15. A wife should have an independent personality and not always obey her husband. □ 1 □ 2 □ 3 □ 4 □ 5 □ 6

**End of Questionnaire — Thank you very much for your participation!**
